# Ionospheric monitoring with the Chilean GPS eyeball during the South American total solar eclipse on 2nd July 2019

**DOI:** 10.1038/s41598-020-75986-7

**Published:** 2020-11-09

**Authors:** Ajeet K. Maurya, Mahesh N. Shrivastava, Kondapalli Niranjan Kumar

**Affiliations:** 1 Department of Physics, Doon University, Dehra Dun, India; 2 Universidad Católica del Norte, Antofagasta, Chile; 3 National Research Center for Integrated Natural Disaster Management, Santiago, Chile; 4 National Centre for Medium Range Weather Forecasting, Ministry of Earth Sciences, Noida, India

**Keywords:** Solar physics, Atmospheric dynamics

## Abstract

The impact of total solar eclipse of July 2, 2019 on the Ionosphere is studied using 24 Chilean GPS stations north–south of the totality path. The total solar eclipse passed through Coquimbo region from ~ 16:38 CLT (~ 20:38 UTC) to ~ 16:40 CLT (~ 20:40 UTC) and maximum eclipse was observed ~ 16:39 CLT (~ 20:39 UTC). The total electron content (TEC) derived from GPS signals shows peculiar features. At the totality stations TEC variations are small (~ 0.39 TECu), but it shows significant decrease (maximum ~ 2.24 TECu) for stations located south and increase (maximum ~ 3.89 TECu) for the stations located north of totality of the surface. The wavelet analysis of VTEC timeseries shows the presence of strong atmospheric gravity waves (AGWs) of duration ~ 30 to 60 min at the stations located north of totality. Thus, the results suggest an interplay between eclipse effect on the ionosphere plasma density and eclipse generated AGWs induced plasma density perturbation provided the peculiar features.

## Introduction

Solar eclipses provide a specific opportunity to assess the ionospheric response during the sudden change in the solar flux. Therefore, it has become widely studied phenomenon with several publications^1–6^. Despite all significant previous work and findings, the study of solar eclipse effect on earth’s atmosphere remains imperative as each eclipse is different in terms of eclipse duration, location, time of the day, season and region of the atmosphere. The total solar eclipse which took place, when the moon moved into a position of direct alignment with the sun and the earth and thus completely blocks the solar disk, causing almost night like conditions for a very short time. Consequently, interrupting photoionization and thermospheric heating, which leads to a disturbance on both the thermosphere and the ionosphere, including a modification of the temperature balance, production, and loss of ionization and generation of atmospheric gravity waves^7–10^. The production and reduction of ionization also depend on the local time. The spatial and temporal variations of the induced ionospheric changes are correlated with the obscuration function of solar radiation. It is also depending on the delayed response to the obscuration function, increasing with the altitude of the ionospheric layer^10^.

The radio remote sensing techniques emerged as a prominent cost-effective tool for continuous monitoring of ionospheric changes due to solar eclipse. The Global Positing System (GPS) derived Total Electron Content (TEC) emerges as one of the widely used radio remote sensing method to monitor ionospheric changes due to a solar eclipse^11–15^. When GPS signals travel through the ionosphere, the navigation signals are delayed due to interaction with ionospheric plasma. The ionospheric propagation delay is directly proportional to TEC. Tsai and Liu^11^ have used five GPS stations and analyze TEC data for 23 October 1999 and 9 March 1999 solar eclipse over the equatorial region and reported decease in TEC during the eclipse. Afraimovich et al.^12^ have also analyzed GPS-derived TEC during the 11 March 1997 solar eclipse and have reported depression depth of TEC ~ 1 to 3 TEC unit. Whereas, Liu et al.^16^ observed ~ 40 to 50% depression in TEC during the 24 October 1995 solar eclipse in the equatorial anomaly region. Apart from the decrease in TEC, several works have also demonstrated the generation of atmospheric gravity waves during the solar eclipse which can severely impact the performance of satellite-based radio-telecommunication and navigation systems^9,15,17^.

**Figure 1:**
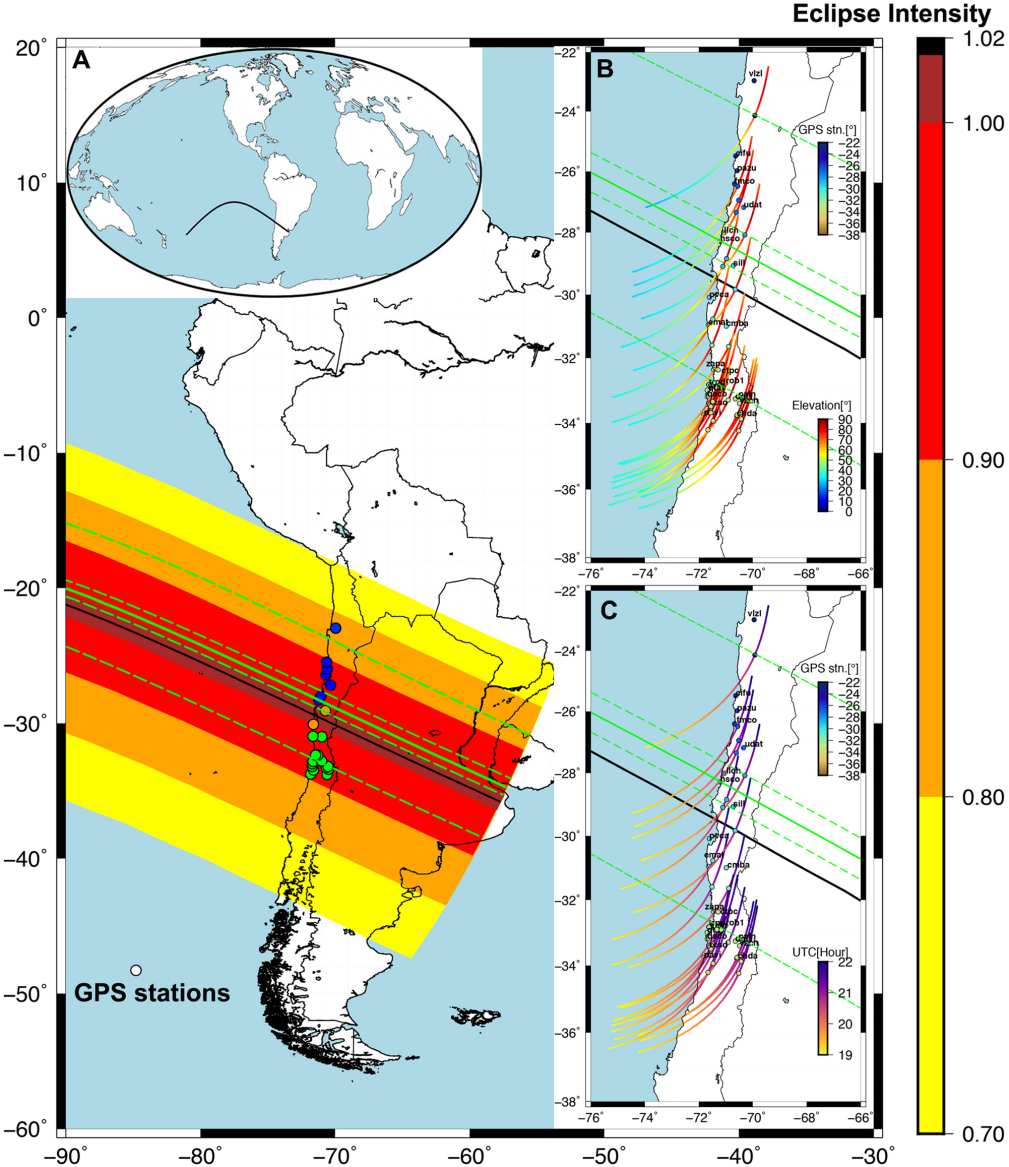
The geographical map of the South American region. The different color circles are showing the GPS stations used in the current analysis. The GPS stations following in the totality region on the surface are plotted with orange color circles, north of the totality in the blue color circles and south of the totality in the green color circles. The black line shows the eclipse central line. The green line shows the eclipse central line at altitude 350 km and dotted lines are 100%(1.00) and 90%(0.90) of the eclipse totality. Inset (**A**) the path of the total solar eclipse central line on the world map. (**B**) The elevation of GPS satellites path at 350 km altitude with respect to ground GPS stations with color table from blue to red and IPP represent on the path with color circles. The color circles of GPS stations are plotted with respect to latitude. (**C**) The UTC of GPS satellites path at 350 km altitude with color table from yellow to violet. The color of GPS stations is with respect to latitude. The figure is prepared using the (URL: https://gmt.soest.hawaii.edu/projects/gmt) Generic Mapping Tools (GMT)^37^ 5.1.1.

The total solar eclipse occurred at the ascending node of the Moon's orbit on July 2, 2019, with an eclipse magnitude of 1.023. The totality of solar eclipse was visible from the southern Pacific Ocean, east of New Zealand to the Coquimbo Region in Chile and Central Argentina. The maximum totality 4 min 32 s visible from the Pacific Ocean. More information about this eclipse can be found at (https://eclipse.gsfc.nasa.gov/SEgoogle/SEgoogle2001/SE2019Jul02Tgoogle.html). The total solar eclipse occurred in the Coquimbo region from 16:38:18 to 16:40:54 Chilean local Time (CLT = UTC—4:00:00) around 2 m 36 s, moving from North-East towards South-West. However, the total solar eclipse time in the Coquimbo region was from 15:22:20 to 17:46:30 CLT around 2 h 24 m 10 s. The maximum total eclipse was at 16:39:12 CLT. Outside of the totality zone, the shadow of the eclipse was still visible to varying degrees across the continent (see Fig. 1). In this paper, we provide a detailed analysis of GPS TEC variations during July 2, 2019 total solar eclipse by the GPS stations located both sides (north and south) of the totality path, thus providing latitudinal variation. We have observed peculiar features in TEC for the different GPS stations not reported before. The observations reported here show an important role of eclipse generated AGWs and background wind direction in affecting GPS signals and thus TEC.

## Methodology: total electron content (TEC) from GPS data

The total solar eclipse occurred on July 2, 2019 and its details are shown in Fig. 1 and in its subsections. The eclipse conditions at the ground levels are shown in Fig. 1 as color coded with 100% to 70% eclipse obscuration. The central line of the eclipse totality path at ground level is shown by black color. The global path of central totality line at ground level is shown in Fig. 1A. The path of the solar eclipse was from the southern Pacific Ocean, east of New Zealand to the Coquimbo Region in Chile and Central Argentina. We have used a total of 24 stations, which lies in the totality as well as up to ~ 80% solar obscuration both sides of the totality line on the surface. There were 2 GPS sites on the path of the total solar eclipse (pvca & sill) shown with brown color filled circles, 15 sites south of totality shown with green color filled circles and 7 sites north of totality shown with blue color filled circles. More details about the GPS stations and eclipse conditions (with respect to ground level) can be found in Table 1 and supporting Table S1. Further, we have also estimated eclipse conditions at the ionospheric height of 350 km by the method suggested by Verhulst et al.^18^ and shown with green lines (totality central line as solid green line, totality north, south limit, and 90% obscuration lines as dashed green lines).

**Table 1:** Detail about the GPS stations analyzed, their location, eclipse condition, magnitude and change in VTEC at each station.

S. no.	GPS station	Lat (S)	Long. (W)	Eclipse start time (UTC)	Max eclipse time (UTC)	Eclipse end time (UTC)	%Obscuration (magnitude)	Change in VTEC in TEC unit
1	vlzl	− 22.96	− 69.95	19.49	20.75	21.83	79.5 (0.83)	− 3.39
2	cifu	− 25.48	− 70.63	19.44	20.72	21.82	89.4 (0.91)	− 3.89
3	pazu	− 25.97	− 70.58	19.44	20.71	21.82	90.7 (0.92)	− 3.87
4	fmco	− 26.4	− 70.67	19.43	20.7	21.82	92.7 (0.94)	− 3.16
5	udat	− 27.18	− 70.33	19.43	20.7	21.82	94.3 (0.95)	− 3.45
6	llch	− 28.01	− 70.06	19.4	20.68	21.8	98.06 (0.98)	− 1.7
7	hsco	− 28.28	− 71.21	19.4	20.675	21.8	98.81 (0.99)	− 1.59
8	sill	− 29.07	− 70.722	19.4	20.67	21.79	100 (1.00)	0.38
9	pvca	− 30.08	− 71.6	19.36	20.65	21.77	100 (1.00)	0.56
10	emat	− 30.96	− 71.646	19.35	20.64	21.76	97.96 (0.98)	1.21
11	cmba	− 31	− 70.98	19.37	20.65	21.76	98.55 (0.98)	2.24
12	zapa	− 32.36	− 71.5	19.35	20.62	21.74	94.28 (0.95)	1.3
13	ctpc	− 32.37	− 71.28	19.34	20.62	21.74	94.28 (0.95)	1.18
14	rob1	− 32.78	− 71	19.34	20.62	21.73	93.7 (0.94)	1.43
15	valn	− 32.83	− 71.61	19.33	20.61	21.73	92.7 (0.94)	0.84
16	trpd	− 32.83	− 71.63	19.34	20.61	21.73	92.7 (0.94)	1.18
17	qtay	− 33	− 71.68	19.33	20.61	21.72	91.9 (0.93)	1.26
18	qsco	− 33.19	− 70.68	19.33	20.6	21.72	91.6 (0.93)	0.89
19	caln	− 33.2	− 70.52	19.37	20.62	21.73	91.6 (0.93)	1.57
20	dgf1	− 33.26	− 70.64	19.36	20.62	21.73	91.6 (0.93)	0.775
21	vzch	− 33.4	− 70.48	19.37	20.61	21.73	90.6 (0.92)	1.33
22	rcsd	− 33.46	− 71.59	19.36	20.6	21.72	90.6 (0.92)	1.19
23	navi	− 33.75	− 71.8	19.36	20.6	21.72	89.6 (0.91)	1.96
24	chda	− 33.79	− 70.59	19.36	20.61	21.72	90.6 (0.91)	1.53

All the GPS receiving stations are of Continuously Operating, managed by the Chilean Centro Sismológico Nacional (CSN). The GPS data can be obtained on request from corresponding author for scientific purpose. All the GPS receivers collect dual-frequency signals operated at L1 (1575.42 MHz) and L2 (1227.60 MHz). The GPS receiver stations receive signals transmitted by satellites orbiting at an altitude of ~ 20,000 km. To get to the earth, these signals just first pass through the ionosphere. The integration of the number of electrons in the ionosphere is proportional to the delay between L1 and L2. The data obtained from each receiver are in the Receiver Independent Exchange Format (RINEX) and processed for TEC format. The format conversion was carried out by using GPS_GOPI software (https://seemala.blogspot.com/2017/09/gps-tec-program-ver-295.html) to obtain the TEC parameters^19,20^. The vertical TEC (VTEC) used in this work is estimated from the line-of-sight TEC (STEC) values using a simple mapping function and are associated to an ionospheric pierce point (IPP) latitude and longitude, assuming the ionosphere to be compressed into a thin shell Single Layer Ionosphere Model (SLIM) at the peak ionospheric height of 350 km^21^.

In this study analyzed VTEC is based on data recorded on July 1st, 2nd and 3rd in 2019 from 18:00:00 UTC until 23:00:00 UTC before and after the total solar eclipse. The data were filtered to include only signals from all satellites having elevation angle > 20°. The filtering was carried out to avoid disturbances such as multipath, noises caused by high-rise buildings or tall trees, or other causes that are not derived from the effects of the ionosphere. Geographically each station was located at different coordinates so they sense different eclipse obscuration. We analyze the vertical TEC (VTEC) data from all 24 GPS stations corresponding to the pseudo-random number (PRN) 13. The detailed path of PRN13 at IPPs color coded with reference to elevation angle (in degree) and time (in UTC) is shown in Fig. 1B, C respectively. As per the Fig. 1B, VTEC during eclipse time (~ 19:30–22:00 UTC), corresponds to the elevation angle ~  > 50°. Figure 1C, show PRN 13 have wide time coverage before and after the solar eclipse for all utilized GPS stations.

### VTEC analysis results

**Figure 2:**
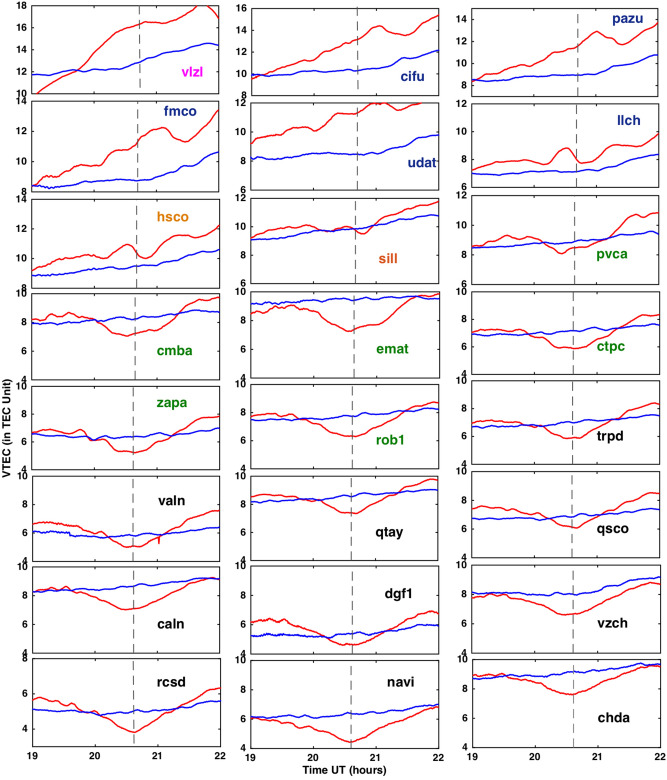
Show VTEC time series derived from the 24 GPS stations. The red plot is the VTEC on eclipse day (July, 2, 2019), while blue plot (called unperturbed plot) is the mean of two days (July 1 and 3, 2019) for PRN 13. The plots are arranged from bottom (South) to the top (North). The GPS site name in each subplot is color coded with eclipse obscuration at 350 km height. The totality stations (‘sill’ and ‘hsco’) are shown in brown color, stations within 90% eclipse and in north of totality line shown in blue color, stations with < 90% eclipse in north shown in magenta color, stations within 90% totality and located south of totality line shown in green color, and stations with < 90 eclipse in south shown in black color. The grey dotted vertical line is showing maximum eclipse time at each station. The figure is prepared using the (https://gmt.soest.hawaii.edu/projects/gmt) GMT^37^ 5.1.1.

We have used VTEC data for each station for three days: eclipse day (July 2, 2019), the day before the eclipse (July 1, 2019) and the day after the eclipse (July 3, 2019). The mean (unperturbed) VTEC is estimated from two days VTEC (July 1 and 3, 2019). The final results are presented in Fig. 2. Figure 2 shows the VTEC variation as a function of time for the unperturbed day (blue) and eclipse day (red) and plotted for each station. Each plot is shown for 3 h duration (i.e. 19–22 UTC) which corresponds to the eclipse duration over the region. The vertical line on each plot shows the maximum eclipse time. The GPS site name in each subplot in Fig. 2 color coded to show the percentage of eclipse obscuration at 350 km height. The totality stations (‘sill’ and ‘hsco’) are shown in brown color, stations within 90% eclipse and located north of totality line shown in blue color, stations with < 90% eclipse in north shown in magenta color, stations within 90% totality and located south of totality line shown in green color, and stations with < 90 eclipse in south shown in black color.

As shown in Table 1, the eclipse start time for all stations on average ~ 19.35 UTC (15.35 CLT) h and end time ~ 21.72 UTC (17.72 CLT) h, and maximum eclipse occurred ~ 20.60 UTC (16.60 CLT) h, which correspond to late afternoon to evening time at the stations. This is the time when ionospheric electron density is subsidizing due to a drop in solar ionizing radiation, thus provide a unique opportunity to study ionosphere under decreasing radiation conditions. Another interesting observation from Fig. 2, all the time series of VTEC in the 3 days showing an increasing trend. Though for the stations in the south, the trend is less pronounced as compared to stations in the north. This suggests that, probably towards the north, VTEC increasing. To better understand this, we have looked into the PRN13 path with time (Fig. 1C), which shows satellite propagating towards the north with time. Thus, this is possible that north of GPS station there is an increase in VTEC. However, at present, the clear reason for such an increase in VTEC is not known. Therefore, a thorough study of diurnal variation of VTEC with long term data set is needed to clearly understand such variations. We have planned this as a separate future study. Further, it can be seen from Fig. 2, on an average change in VTEC started ~ 20 UTC after ~ 30 min of start of the eclipse and recovered ~ 21.5 UTC, ~ 20 min before the end of the eclipse at the stations. Thus, there is a delay in TEC response to the solar eclipse, which is very well reported in many previous works and also verified in present work^13,14^.

## Discussion

The most important peculiar feature of the present observation is different VTEC variation at different stations with reference to the central totality line (green solid line in Fig. 1) at 350 km height. At the ‘sill’ which is totality stations (located south of central totality green line), there is a decrease in VTEC during the eclipse which is ~ 0.38 TECu with reference to an unperturbed value close to maximum eclipse time. As one moves south of the central totality green line, the depression depth of VTEC on eclipse day compared to the unperturbed value becomes higher. At ‘hsco’ which is a totality station (located north of central totality green line), there is an increase in VTEC during the eclipse with ~ 1.59 TECu with reference to an unperturbed value close to maximum eclipse time. As one moves north of the central totality path (green solid line), VTEC on eclipse day is further increases with reference to the unperturbed value. Thus, at totality stations, VTEC difference with reference to the unperturbed value is minimum and increases as one moves away from the totality central line (green solid line). Such kind of peculiar changes in VTEC during a solar eclipse as per the author’s knowledge is reported for the first time. As shown in Table 1, the maximum decrease in VTEC ~ 2.24 TECu at ‘cmba’ station and maximum increase in VTEC ~ 3.89 TECu at ‘cifu’ station as compared to an unperturbed value close to maximum eclipse time.

It is important to discuss space weather (solar flare and geomagnetic storm) conditions as these are important contributors of VTEC variability^22,23^. We have verified from https://www.spaceweatherlive.com/ and found that there was no solar flare event occurred during three days (1–3 July 2019) that can affect the ionosphere. The geomagnetic data is downloaded from world data center Kyoto Japan (https://wdc.kugi.kyoto-u.ac.jp/). We have checked three hourly planetary K index (K_p_) and disturbance storm time index (Dst) providing the conditions for the geomagnetic activity for three days during 1–3 July 2019. The Maximum K_p_ index was + 3 on 1st July during 03 UTC. The minimum Dst was ~ -18 nT at 22 UTC on 1st July 2019. These values show no geomagnetic activity. Thus, there was no magnetospheric input during the analysis period i.e. 1–3 July 2019.

**Figure 3:**
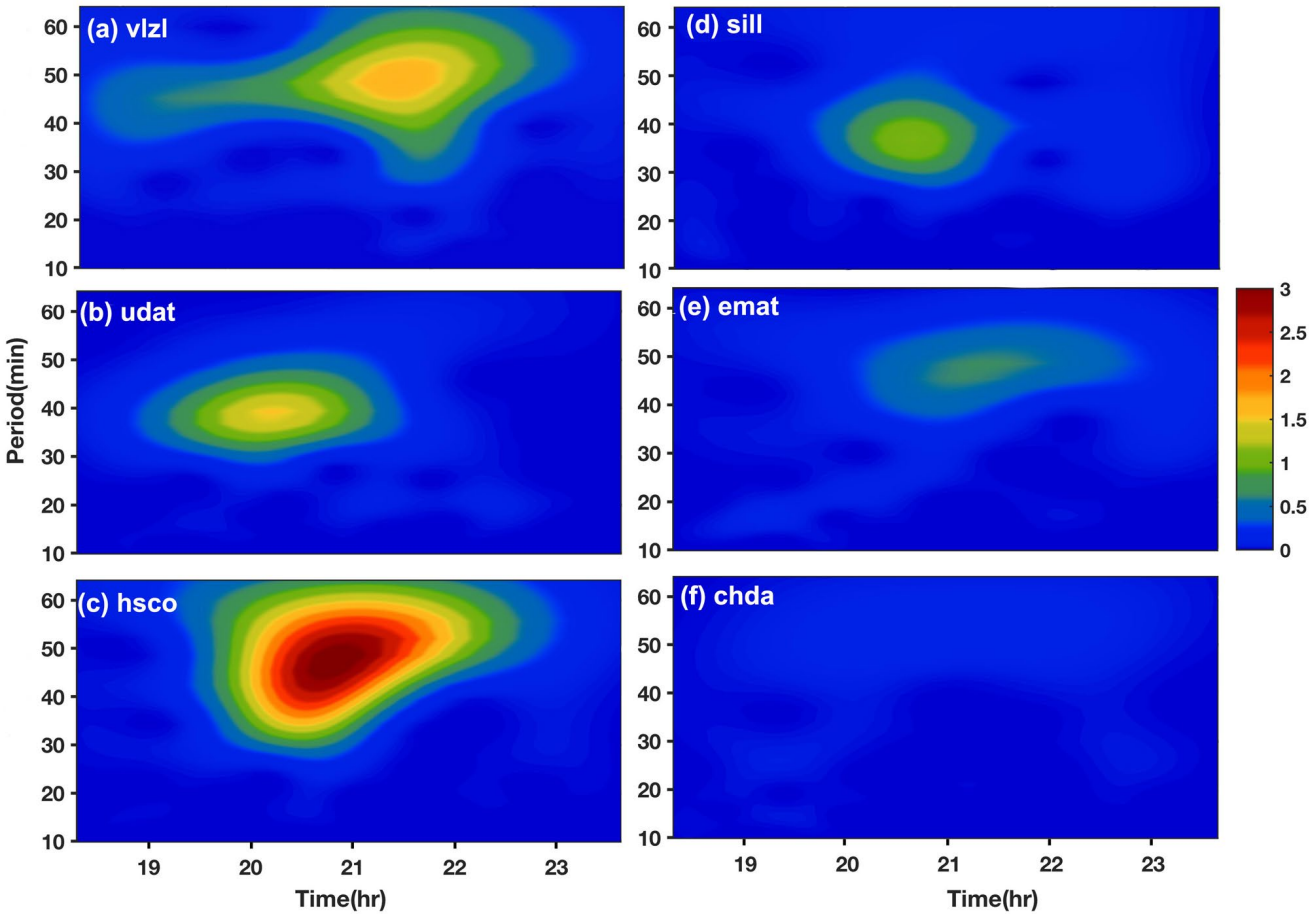
Wavelet analysis of VTEC time series using Morlet mother wavelet, show atmospheric gravity waves with period ~ 30 to 60 min are present during eclipse. Six representative stations are shown, (**a**) station at far north, (**b**) station close to totality, (**c**) in totality, (**d**) in totality (**e**) close to totality in north, (**f**) station at far south. The figure is prepared using the (https://gmt.soest.hawaii.edu/projects/gmt) GMT^37^ 5.1.1.

In order to understand reported peculiar observations in this work, we have looked into the role of atmospheric gravity waves (AGWs) generated during the solar eclipse. As suggested by Chimonas and Hines^2^, during solar eclipse moon blocks the direct UV radiation coming from the Sun, thus ozone heating stops in the stratosphere and creates a cooling spot in the stratosphere which moves with the supersonic speed across the Earth and acts as a source of disturbance that creates AGWs. To see the presence of AGWs, we have analyzed VTEC data using the Morlet wavelet analysis technique as discussed in many of the previous works^4,24,25^. We did wavelet analysis for all 24 stations but the wavelet plot for only six representative stations is shown as Fig. 3. For the analysis, first, we have filtered fluctuations greater than 2 h to see the high-frequency fluctuations caused by the eclipse. For the filtered time series, we performed the Morlet wavelet analysis for the station running from south to north. In Fig. 3, wavelet analysis for stations in totality region with reference to green solid line (sill, hsco), partial eclipse region (emat, udat, > 90% eclipse obscuration) and stations located extreme south and north (chda, vlzl, < 90% eclipse obscuration) is shown. As one can see Fig. 3f ‘chda’ station located extreme south (< 90% obscuration) there is no AGWs seen, in Fig. 3e ‘emat’ station (within 90% obscuration) south of totality line AGWs with period ~ 40 to 60 min started appearing but are of very low intensity, in Fig. 3d ‘sill’ station, (totality station but south of central line) AGWs intensity increases also a slight change in periodicity (~ 30 to 50 min), in Fig. 3c ‘hsco’ station (totality station but north of central line) show AGWs with ~ 30 to 60 min with very high intensity, in Fig. 3b, ‘udat’ station (within 90% obscuration) north of totality line AGWs with period ~ 30 to 50 min appeared but the intensity is lower compared to ‘hsco’ station. in Fig. 3a, ‘vlzl’ station (< 90% obscuration) located extreme north, AGWs with a similar period are present but of reduced intensity. It is important to note that AGWs are seen after ~ 20 UTC at each station, which ~ 30 min after the start of the eclipse, thus most probably generated by the eclipse.

Further, as AGWs saw up to at ‘emat’ station in the south with weak intensity, and are present at all stations in the north with strong intensity, which suggests that AGWs generated during the solar eclipse, propagates towards north and south of totality line, but due to some reasons, AGWs propagation towards the south is restricted, thus the weak AGWs are seen only at the stations close to totality. As strong AGWs are seen at the stations located north of totality, this is suggested that some background conditions probably forcing AGWs to propagate northward thus we see strong wave signature at the stations located north of totality line.

**Figure 4:**
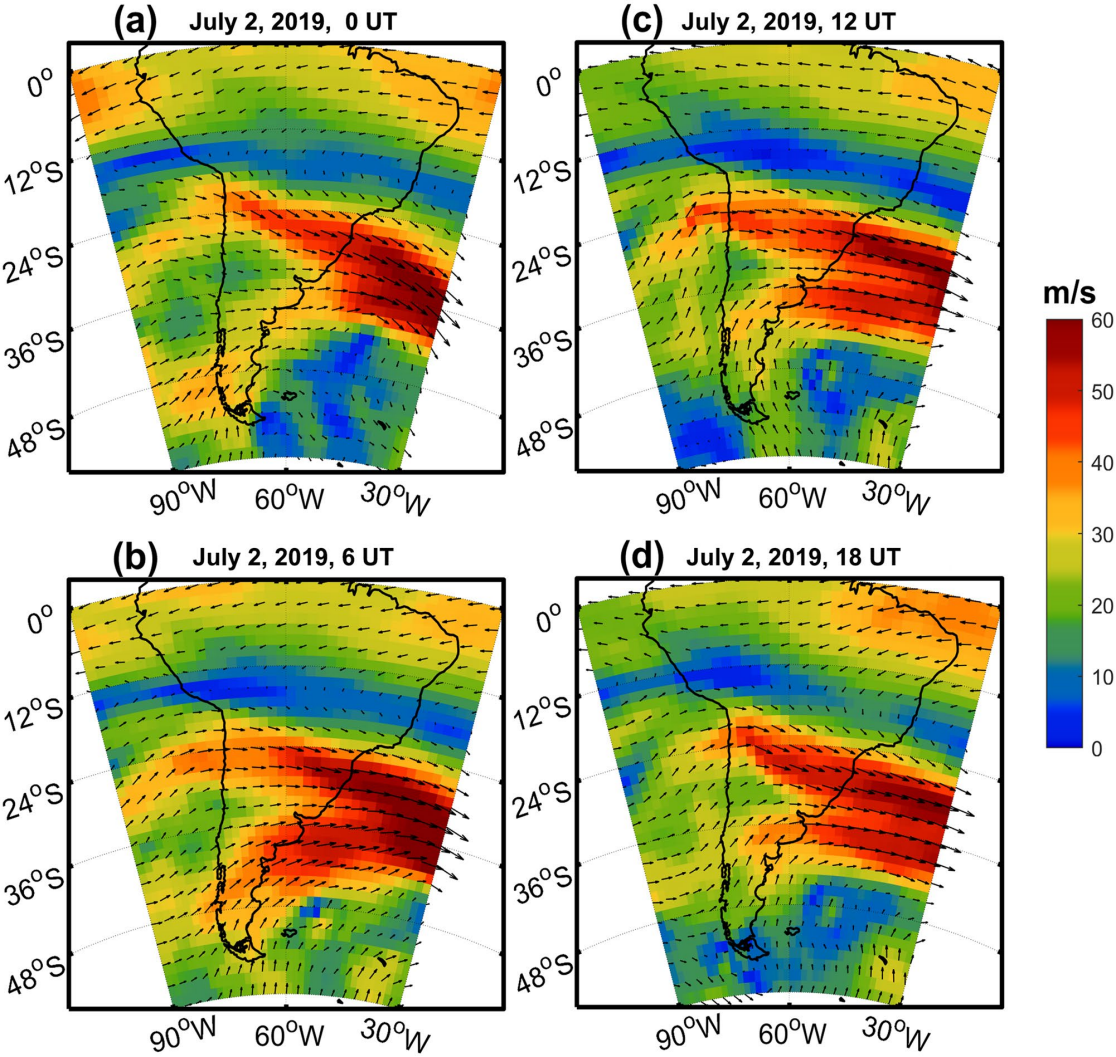
The background wind speed (shaded) and vector winds over the South America on for four different timings (0, 6, 12 and 18 UTC) on solar eclipse day on July 2, 2019 at ~ 80 km from ERA5 reanalysis data. The figure is prepared using the (https://gmt.soest.hawaii.edu/projects/gmt) GMT^37^ 5.1.1.

In order to understand the background conditions affecting AGWs propagation, we have analyzed the background wind data and presented in Fig. 4. The wind data is used from the European Centre for Medium‐Range Weather Forecasts (ECMWF) Reanalysis 5 product *ECMWF*^26^. The data are produced in 137 hybrid sigma pressure levels covering the surface to ~ 80 km altitude, with a spatial resolution of ~ 31 km^27^, which can be downloaded from the following link (https://cds.climate.copernicus.eu/). Figure 4, shows the background wind speed (shaded) and vector winds over South America for four different timings (0, 6, 12 and 18 UTC) on solar eclipse day on July 2, 2019 at ~ 80 km. The basic winds are westerlies all over the day. However, from 6 UTC the winds are south-westerlies to the south of Chile region. The south-westerlies are more prominent during peak hours of the solar eclipse. At the same time the northern part of Chile, the winds are westerlies during the initial hours of the day while they become north westerlies during peak hours of the day. The winds are converging from south and north of the Chile region and they strengthened with magnitudes of about 60 m/s to the east. As suggested in previous works^28–30^ the AGWs can easily pass through the critical levels when they propagate opposite to wind direction. It means that background wind direction during eclipse time supports the AGWs propagation towards northward and opposes southward. The south westerlies continue after the solar eclipse event day until 12UTC on 3 July 2019, however late evening hours after 12 UTC, the wind direction slowly changes to westerlies (Supplementary figure S1). Thus, AGWs propagating towards north more prominently and southward propagating waves are slowed down/attenuated. Maurya et al.^30^ reported thunderstorm/convection generated AGWs which are propagating opposite to the background wind direction reaching the GPS station and affecting VTEC signals. Nishioka et al.^31^ also discussed the important role of background wind and suggested when background wind velocity is comparable to the phase velocity of AGWs, their propagation along the wind direction are restricted/reduced.

As from the above discussion it is clear that AGWs were generated during the solar eclipse and were propagating toward the north of the totality line due to favorable conditions from the background wind. Therefore, it is important to discuss the role of AGWs in modifying ionospheric electron density, thus causing observed VTEC variations. As suggested by Somsikov^32^, AGWs generated by the solar eclipse propagates upward into the thermosphere-ionosphere system and manifest themselves as plasma density fluctuations, which propagates as a wave, away from the totality. These plasma density fluctuations are also known as traveling ionospheric disturbances (TIDs)^17^. Liu et al.^16^ observed AGWs excited during solar eclipse events at F region altitude and their generation attributed to changes in the height of the peak of electron production. Xinmiao et al.^33^ reported synchronous oscillations in the Es and F layer during the recovery phase of the solar eclipse whereas, Ivanov et al.^34^ observed traveling ionospheric disturbances during a solar eclipse with a period about 40 min. Oscillations in the ionosphere, similar to gravity waves, were observed following some solar eclipse events^2,16,21,35^. Coster et al.^36^ analyzed GNSS TEC data over continental united states during 21 August 2017, total solar eclipse, and reported significant TEC depletion except for station at the Rocky mountain chain region, where they have observed TEC enchantment during the main period of TEC depletion. They suggested the interaction of eclipse-induced mountain waves due to surface changes in pressure and temperature coupled with the eclipse-induced ozone cooling and other wind variations may produce the observed enhanced TEC. In the present case, all the GPS stations are in the coastal region of Chile which is bounded in the east by the great Andes mountain range. Thus, there is a possibility of the presence of eclipse-induced mountain waves due to surface changes in pressure and temperature. Hence interplay between eclipse-induced mountain waves due to surface changes in pressure and temperature coupled with the eclipse-induced ozone cooling may be causing an observed enhancement in VTEC. Though, due to the lack of suitable observation facilities such as Radar/Lidar, we cannot verify the presence or absence of eclipse-induced mountain waves.

In present observations at totality stations, we have observed minimum change in TEC compared to stations located in partial eclipse region. It is well known that maximum change is observed at the eclipse totality and effect decreases as one moves from the totality path. Although not very well understood but the most probable reason could be interplay between eclipse induced reduction in electron density and eclipse generated AGWs induced electron density variations. Further, as there is significant electron density reduction at the stations located south of totality, and increase in electron density for stations north of totality line. Thus, the eclipse waves may be generated like the ship wakes. Hence, they generated at both sides but southward propagating waves are indeed filtered out due to the background winds while the northern side of the eclipse background is conducive for their propagation. Therefore, decreasing electron density at the stations at the south and increasing electron density at the stations in the north.

## Summary

On July 2, 2019, a total solar eclipse occurred over the South American region, during evening time (~ 15:20 CLT-17:50 CLT) along a path from the southern Pacific Ocean, east of New Zealand to the Coquimbo Region in Chile and Central Argentina. The partial eclipse was observed in a much larger region of southern America. In order to understand the eclipse effect on the ionosphere, data from 24 GPS stations located across the totality path were analyzed. The VTEC derived from GPS TEC signal for 24 stations during eclipse showed peculiar features not reported before. The VTEC time series variation at the totality station is small, whereas it shows a significant decrease (maximum value ~ 2.24 TECu) compare to unperturbed value for stations located south of totality path, and increase (maximum value ~ 3.89 TECu) for the station located north of totality path. Further, to understand these peculiar variations, the wavelet analysis of VTEC time series data was performed, which showed the presence of strong atmospheric gravity waves (AGWs) with period ~ 30 to 60 min at the station located north of totality. The AGWs generated by the solar eclipse propagating north ward supported by the background wind data, which showed south-westerlies during peak hours of the solar eclipse. Overall, the analysis suggests an interplay between direct eclipse effect on the ionosphere plasma density and eclipse generated AGWs induced plasma density perturbation for the observed peculiar features.

## Supplementary information

Supplementary Information.
