# Supplementary material for: Ionospheric monitoring with the Chilean GPS eyeball during the South American total solar eclipse on 2nd July 2019
Source: Sci Rep. 2020 Nov 9;10:19380. doi: 10.1038/s41598-020-75986-7 (PMC7652920; doi:10.1038/s41598-020-75986-7)
Supplement: Supplementary file 1 — Supplementary Information. [file 41598_2020_75986_MOESM1_ESM.docx]

­­Supporting Information for

**Ionospheric monitoring with the Chilean GPS eyeball during the South American Total solar eclipse on 2^nd^ July 2019**

Ajeet K Maurya^1^, Mahesh N Shrivastava^2,3*^ and Kondapalli Niranjan Kumar^4^

^1^Department of Physics, Doon University, Dehradun, India

^2^Universidad Católica del Norte, Antofagasta, Chile

^3^National Research Center for Integrated Natural Disaster Management, Santiago, Chile

^4^National Centre for Medium Range Weather Forecasting, Ministry of Earth Sciences, Noida, India.

**Contents of this file**

Table S1

Figure S1

**Introduction**

This supporting material consists of one supporting table of the GPS stations and one supporting figure of background wind speed used for this study.

**Table S1**: Detail about the GPS stations analyzed, their location, eclipse condition, magnitude and change in VTEC at each station

| **S.N.** | **GPS Station** | **Lat.**  **(^0^S)** | **Long. (^0^W)** | **Eclipse Start time [UTC]** | **Max eclipse time [UTC]** | **Eclipse End time [UTC]** | **Eclipse Magnitude** | **Change in VTEC in TEC unit** |  |
| --- | --- | --- | --- | --- | --- | --- | --- | --- | --- |
| 1 | chda | -33.79 | -70.59 | 19.36 | 20.61 | 21.72 | 0.91 | 1.53 |  |
| 2 | navi | -33.75 | -71.8 | 19.36 | 20.6 | 21.72 | 0.91 | 1.96 |  |
| 3 | rcsd | -33.46 | -71.59 | 19.36 | 20.6 | 21.72 | 0.92 | 1.19 |  |
| 4 | vzch | -33.4 | -70.48 | 19.37 | 20.61 | 21.73 | 0.92 | 1.33 |  |
| 5 | dgf1 | -33.26 | -70.64 | 19.36 | 20.62 | 21.73 | 0.92 | 0.775 |  |
| 6 | caln | -33.2 | -70.52 | 19.37 | 20.62 | 21.73 | 0.92 | 1.57 |  |
| 7 | qsco | -33.19 | -70.68 | 19.33 | 20.6 | 21.72 | 0.92 | 0.89 |  |
| 8 | qtay | -33 | -71.68 | 19.33 | 20.61 | 21.72 | 0.92 | 1.26 |  |
| 9 | valn | -32.83 | -71.61 | 19.33 | 20.61 | 21.73 | 0.93 | 0.84 |  |
| 10 | trpd | -32.83 | -71.63 | 19.34 | 20.61 | 21.73 | 0.93 | 1.18 |  |
| 11 | rob1 | -32.78 | -71 | 19.34 | 20.62 | 21.73 | 0.94 | 1.43 |  |
| 12 | ctpc | -32.37 | -71.28 | 19.34 | 20.62 | 21.74 | 0.94 | 1.18 |  |
| 13 | zapa | -32.36 | -71.5 | 19.35 | 20.62 | 21.74 | 0.94 | 1.3 |  |
| 14 | cmba | -31 | -70.98 | 19.37 | 20.65 | 21.76 | 0.98 | 2.24 |  |
| 15 | emat | -30.96 | -71.646 | 19.35 | 20.64 | 21.76 | 0.97 | 1.21 |  |
| 16 | pvca | -30.08 | -71.6 | 19.36 | 20.65 | 21.77 | 1 | 0.56 |  |
| 17 | sill | -29.07 | -70.722 | 19.4 | 20.67 | 21.79 | 1 | 0.38 |  |
| 18 | hsco | -28.28 | -71.21 | 19.4 | 20.675 | 21.8 | 0.98 | -1.59 |  |
| 19 | llch | -28.01 | -70.06 | 19.4 | 20.68 | 21.8 | 0.97 | -1.7 |  |
| 20 | udat | -27.18 | -70.33 | 19.43 | 20.7 | 21.82 | 0.94 | -3.45 |  |
| 21 | fmco | -26.4 | -70.67 | 19.43 | 20.7 | 21.82 | 0.93 | -3.16 |  |
| 22 | pazu | -25.97 | -70.58 | 19.44 | 20.71 | 21.82 | 0.92 | -3.87 |  |
| 23 | cifu | -25.48 | -70.63 | 19.44 | 20.72 | 21.82 | 0.9 | -3.89 |  |
| 24 | vlzl | -22.96 | -69.95 | 19.49 | 20.75 | 21.83 | 0.83 | -3.39 |  |


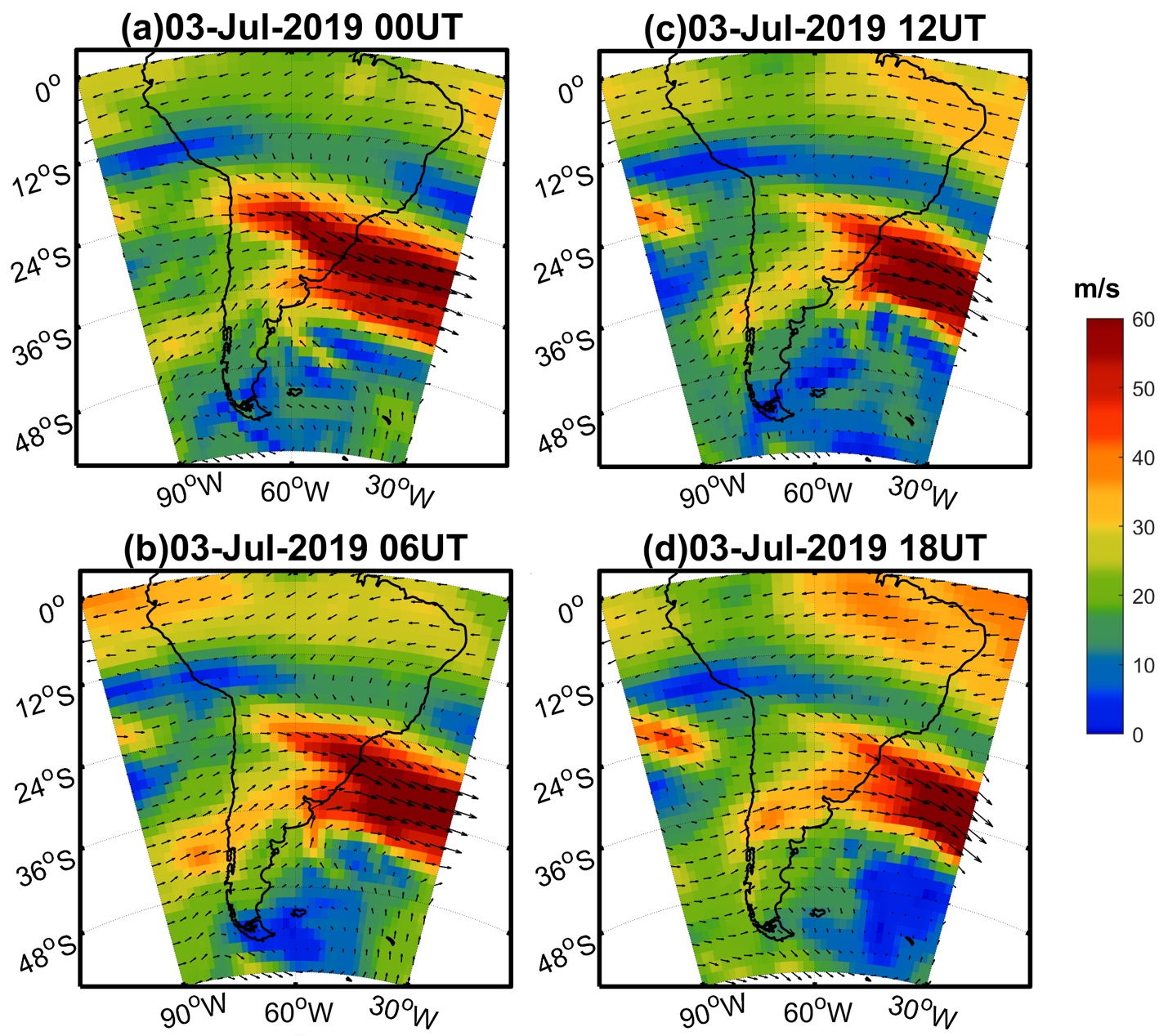


**Figure S1:** The background wind speed (shaded) and vector winds over the South America on for four different timings (0, 6, 12 and 18 UTC) on solar eclipse day on July 3, 2019 at ~80 km from ERA5 reanalysis data. The figure is prepared using the (URL: <http://gmt.soest.hawaii.edu/projects/gmt>) GMT 5.1.1^1^.

**References**

1. Wessel, Paul, et al. Generic mapping tools: improved version released. *Eos, Transactions American Geophysical Union* 94.45: 409-410. https://doi.org/10.1002/2013EO450001 (2013).
